# The gut mycobiome signatures in long-lived populations

**DOI:** 10.1016/j.isci.2024.110412

**Published:** 2024-06-28

**Authors:** Lixia Pu, Shifu Pang, Wenjie Mu, Xiaodong Chen, Yang Zou, Yugui Wang, Yingying Ding, Qi Yan, Yu Huang, Xiaochun Chen, Tao Peng, Weifei Luo, Shuai Wang

**Affiliations:** 1 State Key Laboratory for Animal Disease Control and Prevention, College of Veterinary Medicine, Lanzhou University, Lanzhou Veterinary Research Institute, Chinese Academy of Agricultural Sciences, Lanzhou, Gansu, China; 2 AIage Life Science Corporation Ltd., Guangxi Free Trade Zone Aisheng Biotechnology Corporation Ltd., Nanning, Guangxi, China; 3 Guangxi Key Laboratory of Longevity Science and Technology, AIage Life Science Corporation Ltd., Nanning, Guangxi, China; 4 Guangxi Key Laboratory of Enhanced Recovery After Surgery for Gastrointestinal Cancer, Department of Hepatobiliary Surgery, The First Affiliated Hospital of Guangxi Medical University, Nanning, Guangxi, China

**Keywords:** age, human, microbiome, mycology

## Abstract

Long-lived individuals have been extensively studied as a model to investigate the role of the gut microbiota in aging, but their gut fungi remain almost unexplored. Here, we recruited a community-dwelling cohort of 251 participants (24–108 years, including 47 centenarians) from Guangxi in China to characterize the gut mycobiome signatures. We found gut mycobiome markedly varied during aging and determined aging as a predominant factor driving these variations. For long-lived individuals, core taxa, including *Penicillium* and *Aspergillus*, were maintained and *Candida* enterotype was enriched when compared with old counterparts. Individuals with this enterotype were more likely to possess *Bacteroides* enterotype enriched in young and centenarians. Moreover, the drivers from *Candida* enterotype were positively linked with the bacteria components dominated in *Bacteroides* enterotype. We also identified potentially beneficial yeasts-enriched features to differentiate long-lived individuals from others. Our findings suggest that the gut mycobiome develops with aging, and long-lived individuals possess unique fungal signatures.

## Highlights

• Aging is a major factor affecting the gut fungal variations
• Long-lived individuals have high core fungi levels and enriched *Candida* enterotype
• *Candida* enterotype is linked with *Bacteroides* enterotype for long-lived individuals
• Gut fungal features distinguish long-lived individuals from others

## Graphical abstract

**Figure:**
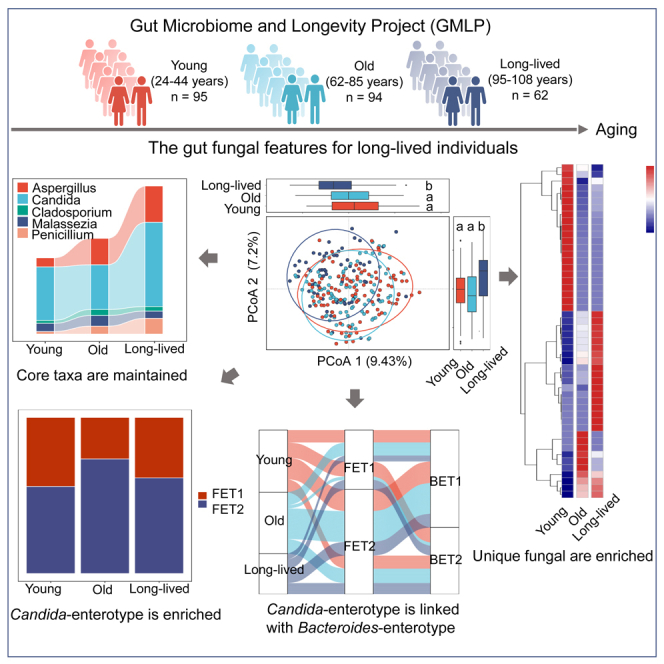

## Introduction

The human gastrointestinal tract hosts a diverse array of microorganisms, encompassing bacteria, viruses, fungi, and eukaryotes, all of which play pivotal roles in the health and disease of the host.^1^^,^^2^^,^^3^ Although the human fungal community, also referred to as the gut mycobiome, constitutes less than 0.1% of the entire human gut microbiome, mounting evidence demonstrates its association with intestinal equilibrium and disease development.^4^ For example, dysregulation of the gut mycobiota has been implicated in multiple human ailments such as hepatobiliary disorders, inflammatory bowel disease (IBD), colorectal cancer, and invasive infections.^5^^,^^6^^,^^7^^,^^8^^,^^9^^,^^10^ Furthermore, the colonization and proliferation of opportunistic fungal pathogens in the gut can trigger perturbed host immune responses, thereby influencing disease progression.^11^^,^^12^

Recent research across different age groups highlights the potential role of the gut mycobiome in the aging process. Studies suggest that the gut fungal community assembles before birth,^13^ with the gut mycobiome of infants initially dominated by *Saccharomycetales* and *Malasseziales* spp. during the first month, followed by a gradual decline in *Malasseziales* to undetectable levels at 5 months of age.^14^ The transition from breastfeeding to solid food, occurring at 1–2 years of age, marks a critical phase in infant dietary habits, with *Saccharomyces cerevisiae* emerging as the predominant species alongside the appearance of *Cystofilobasidium* spp.*, Ascomycota* spp., and *Monographella* spp.^15^ Subsequent to early childhood, the gut mycobiome continues to evolve and matures into an adult-like fungal community characterized by significantly increased diversity and dominance by the phyla *Ascomycota*, *Basidiomycota*, and *Zygomycota*.^16^ The Human Microbiome Project revealed *Candida*, *Saccharomyces*, and *Cladosporium* are the most abundant genera in healthy adults.^4^ Cross-sectional studies have additionally shown that older individuals (>65 years) exhibit enrichment in genera such as *Penicillium*, *Candida*, *Saccharomyces*, *Malassezia*, and *Aspergillus*, while *Blastobotrys* and *Agaricomycetes* spp. are less prevalent compared to middle-aged adults (45–64 years).^17^^,^^18^ From a diversity standpoint, investigations have identified a decline in fungal diversity in the intestines of individuals aged ≥18 years when compared to infants (0–2 years) and children (3–10 years).^19^ Additionally, a recent study on fungi, encompassing a broad age range (18–89 years), further supports this trend by indicating a decline in fungal alpha diversity with advancing age.^20^ Collectively, these studies provide compelling evidence that human age influences the configuration of the gut mycobiome.

Although these findings have provided insights into the mycobiome features in different age groups, the gut fungal signatures in the context of aging remain poorly characterized. Long-lived individuals such as centenarians (aged >100 years) and nonagenarians (aged 90–100 years), who have successfully navigated the challenges of aging and evaded various chronic diseases, represent an excellent model to study the relationship between aging and gut microbiota.^21^^,^^22^^,^^23^^,^^24^^,^^25^ While it is well-established that the gut bacteria community is closely related to aging and longevity for long-lived populations,^26^ only limited attention has been given to the fungal components in these individuals. To reveal the signatures of the fungal part in the gut microbiota for long-lived cohorts, we present a cross-sectional study of the gut mycobiome for 251 community-dwelling individuals in Guangxi Province, China, spanning different age groups (young, old, long-lived; aged 24–108 years, including 47 centenarians). Our findings revealed that aging is the predominant factor driving variations in the gut mycobiome composition within this cohort. The fungi composition of the long-lived population is characterized by maintained core taxa, enriched *Candida*-dominated enterotype, and increased abundance of potentially beneficial yeasts. These findings provide novel insights into the relationship between aging and the intestinal fungi.

## Results

### Cohort description

To investigate the gut fungal signatures in different age populations, we recruited a cohort of 251 community-dwelling individuals in Guangxi Province, China. This cross-sectional cohort is part of a large cohort “The Gut Microbiome and Longevity Project (GMLP).”^26^ The participants in this study (Table 1) were categorized into three age groups: the long-lived group (ages 95–108 years, *n* = 62, including 47 centenarians; mean = 101), the old group (ages 62–85 years, *n* = 94; mean = 69.9), and the young group (ages 24–44 years, *n* = 95; mean = 34.5). We collected data on the demographic information, lifestyle, and disease of each participant by the questionnaire (Table S1). Among all the recorded factors, sex, body mass index (BMI), smoking, and incidences of high blood pressure are significantly different among these groups (Table 1). All these potential confounding factors were tested and corrected using multivariable association analysis (e.g., MaAsLin2, See STAR methods) throughout this study. To compare the gut mycobiome features for the individuals between the long-lived group and other age groups, the gut fungi were sequenced by targeting the ITS1 region and the taxonomic profiling was performed based on amplicon sequence variant (ASV). After eliminating low-quality reads and chimera, each sample yielded a minimum of 42,032 reads.

**Table 1:** Clinical characteristics of the participants for each aging group

	Young	Old	Long-lived	*P*
	(*n* = 95)	(*n* = 94)	(*n* = 62)	
**Age**				

Mean (SD)	34.5 (4.66)	69.6 (5.53)	101 (3.03)	<0.001
Median [Min, Max]	34.0 [24.0,44.0]	69.9 [62.0,85.0]	100 [95.0.108]	

**Sex, n (%)**				

Male	29 (30.5%)	51 (54.3%)	18 (29.0%)	<0.001
Female	66 (69.5%)	43 (45.7%)	44 (71.0%)	

BMI^a^				

Mean (SD)	22.3 (2.70)	20.9 (2.70)	19.2 (1.99)	<0.001
Median [Min, Max]	22.1 [16.7, 28.9]	20.5 [14.7, 27.5]	19.0 [14.5, 23.9]	

**Smoking status, n (%)**				

Smoker	11 (11.6%)	19 (20.2%)	3 (4.8%)	0.0178
Non-smoker	84 (88.4%)	75 (79.8%)	59 (95.2%)	

**Tea drinking, n (%)**				

Drinker	3 (3.2%)	10 (10.6%)	4 (6.5%)	0.117
Non-drinker	92 (96.8%)	83 (88.3%)	58 (93.5%)	
Missing	0 (0%)	1 (1.1%)	0 (0%)	

**Alcohol drinking, n (%)**				

Drinker	14 (14.7%)	15 (16.0%)	6 (9.7%)	0.52
Non-drinker	81 (85.3%)	79 (84.0%)	56 (90.3%)	

**Hypertension, n (%)**				

Yes	1 (1.1%)	6 (6.4%)	28 (45.2%)	<0.001
No	94 (98.9%)	88 (93.6%)	34 (54.8%)	

Age, Sex, BMI, lifestyle and hypertension data are shown. *p* values indicate significant differences among the three groups. Continuous variables were compared using a two-sided Kruskal-Wallis rank-sum test. A pairwise chi-square test was used to compare categorical variables.

a Body mass Index.

### Aging is a major factor driving gut mycobiome variations

**Figure 1:**
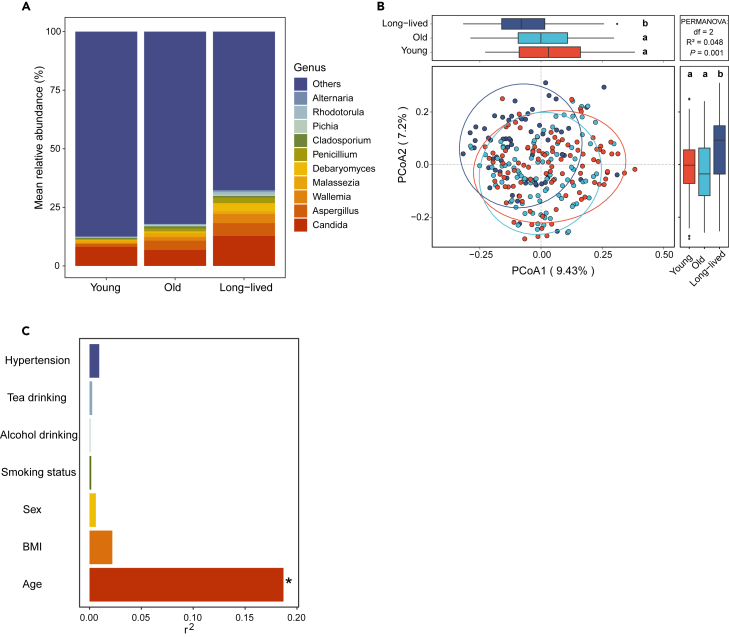
Aging is the major factor affecting the gut mycobiome (A) The mean relative abundance of the top 10 genera. (B) Variation in the gut mycobiome components among groups (*n* = 251) based on Unifrac distances at ASV level (Adonis test, R^2^ = 0.048, *p* = 0.001). Data in boxes with no common letters are significantly different in one-way ANOVA with two-sided Tukey’s post hoc test (*p* < 0.05). (C) The effect size of variables on the gut mycobiome variation at the ASV level. Mycobiome covariates were identified via envfit (vegan). Asterisks indicate the significant covariate (*FDR* < 0.05). BMI, body mass index.

Through taxonomy assignment, 15 gut fungal phyla, encompassing 627 genera at the genus level and 100,190 at the ASV level, were identified in the samples of the three groups. Among them, *Ascomycota* and *Basidiomycota* were the most abundant phyla with a mean relative abundance of 16.12% and 3.21% in the young group, 18.19% and 5.43% in the old group, and 31.53% and 8.33% in the long-lived groups, respectively. At the genus level, *Candida*, *Aspergillus*, *Wallemia*, *Malassezia*, and *Debaryomyces* were the dominant genera, with a mean relative abundance of 8.09%,1.37%,0.10%,1.24%, and 0.32% in young group, 6.76%, 3.40%, 1.54%, 1.61%, 0.85% in the old group, and 12.75%, 5.49%, 3.86%, 1.10%, and 3.55% in the long-lived group, respectively (Figures 1A and S1). To adjust the potential cofounders, we utilized adjusted principal coordinates analysis (aPCoA) based on unweighted UniFrac distance to indicate the compositional variations of the gut mycobiome. This analysis revealed significant shifts in the gut fungal community across different age groups (Figure 1B, *p* = 0.001, R^2^ = 0.0605, PERMANOVA), underscoring variations in gut fungal compositions among the three age groups. In addition, as indicated by PCoA2, the variations observed among age groups (accounting for 7.2% of variations) were not linearly correlated with age. This non-linear development for a small proportion of microbes within the gut mycobiome during aging aligns with previous observations in studies of the gut microbiome^24^^,^^26^^,^^27^ and is consistent with our findings in the gut microbiome of the individuals for this study (Figure S2). As young and long-lived populations generally comprise more females (69.5% and 71.0% in our cohort) than elderly group, we further compared the effect of sex on the mycobiome community and found no significant differences in fungal community structure between males and females (Figure S3).

Gut microbes could be potentially affected by various host factors. To investigate what host factors drive the mycobiome compositions in this cohort, we conducted an envfit analysis based on Bray-Curtis distance to examine the associations between mycobiome and each host variable (including age, BMI, sex, and lifestyle). Remarkably, age is the most effective factor that drives the overall mycobiome variations among these variables (effect size R^2^ = 0.187, *FDR* < 0.05, Table S2) (Figure 1C). Collectively, the results indicate that aging is associated with the gut mycobiome compositions of the studied groups.

### Core taxa are enriched in long-lived individuals

**Figure 2:**
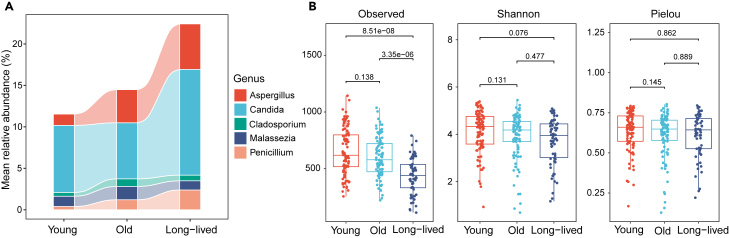
Core taxa are increased in the gut of long-lived individuals (A) The mean relative abundance of core taxa. (B) Comparison of the gut mycobiome α-diversity, as indicated by Observed, Shannon, and Pielou’s evenness indices between groups (*n* = 251). The *p* values in B were calculated by MaAsLin2 with controlled covariates.

To further investigate how aging interacts with the gut mycobiome, we delved into the gut core fungal genera, which are present in at least 85% of the individuals with a minimum relative abundance higher than 0.01%. In accordance with previous studies,^17^^,^^28^^,^^29^ the identified core taxa included *Aspergillus*, *Candida*, *Cladosporium*, *Malassezia*, and *Penicillium* (Figure 2A). Among these taxa, the relative abundance of *Penicillium* and *Aspergillus* displayed significant increases with age (*p* < 0.05; Figure S4), whereas others either remained not altered (*Cladosporium* and *Malassezia*) or exhibited non-linearly changed (*Candida*) during aging. This aging-related trend in the gut mycobiome resulted in increased cumulative relative abundances of these core genera in long-lived individuals, reaching a mean relative abundance of up to 22.42%. These data suggests non-core taxa were depleted for long-lived individuals (Figure 2A). This notion is supported by the species richness analysis in which the species number was significantly decreased in the long-lived group (*p* < 0.05) (Figure 2B). However, Pielou’s evenness as well as the Shannon index was not significantly altered between any age groups (*p* > 0.05). The result suggests that the species’ evenness remains preserved despite a decline in fungal richness during the aging process. Taken together, the previous data pointed to the importance of the core gut fungal taxa in long-lived individuals.

### Fungal enterotypes for long-lived individuals

**Figure 3:**
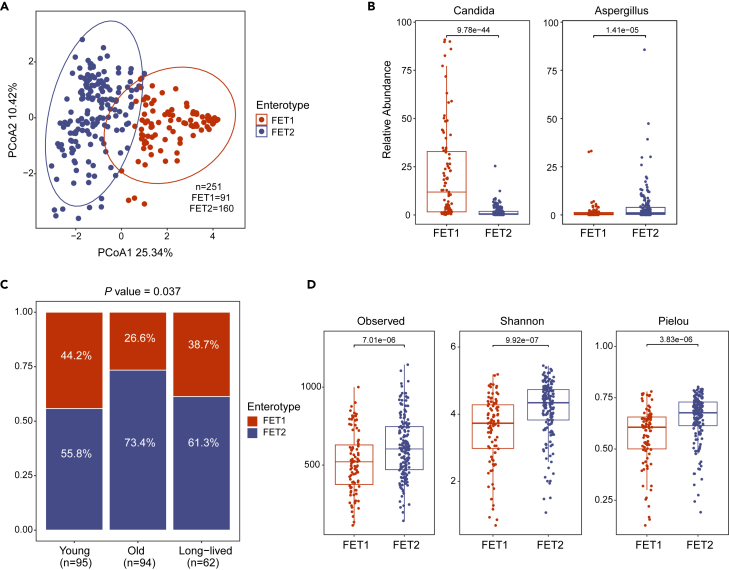
Fungal enterotypes for aging groups (A) The gut mycobiome enterotypes in aging groups. Enterotypes were identified using Jensen–Shannon distance (JSD) and partitioning around medoid (PAM) clustering at the genus level. (B) Relative abundance of the main taxa that drives the clustering within each enterotype. (C) Prevalences of the enterotypes in each aging group. (D) Comparison of α-diversity between two enterotypes. The observed number, Shannon, and Pielou evenness indices are shown. The *p* values in B and D were calculated by MaAsLin2 with controlled covariates. The *p* values in C were calculated by chi-square test.

The gut microbiota profile clusters, also known as enterotypes, have been defined to characterize the bacterial structures in the gut microbiomes.^30^ Herein, we applied this method, using Jensen-Shannon distance (JSD) and partitioning around medoid (PAM) clustering, to compare the fungal composition structure in each age group. For the entire cohort, two enterotypes of the gut mycobiome, designated as fungal enterotype 1 (FET1), and fungal enterotype 2 (FET2) in this study, were identified (Figure 3A). These two enterotypes are distinguishable by the variation in the levels of the genera *Candida* and *Aspergillus*, respectively (Figure 3B). Approximately, 36.3% (*n* = 91) of the gut mycobiome of the subjects belonged to the FET1 (predominantly driven by *Candida*), while the remaining 63.7% (*n* = 160) were categorized under FET2 (nearly equally driven by *Aspergillus*, *Penicillium*, *Wallemia*, and *Malassezia*) (Figure S5). Intriguingly, the proportion of FET1 was much higher in the long-lived group (38.7%) or young group (44.2%) than in the old group (26.6%) (Chi-square test, *p* = 0.037) (Figure 3C). Furthermore, the individuals with the FET1 enterotype exhibited a lower alpha diversity and evenness of the gut mycobiome than those with FET2, as evidenced by the observed ASV number, Shannon, and Pielou indexes (Figure 3D, *p* < 0.05). These data suggests that the fungal enterotypes are associated with gut fungal diversity, in which the *Candida* enterotype exhibited a significantly reduced species diversity. Interestingly, within either the FET1 or FET2 enterotype, the Observed index was significantly decreased along aging groups, similar to the comparisons for the whole cohort whereas Shannon and Pielou’s evenness indices remained relatively stable or mildly altered (Figure S6). These findings suggest that fungal enterotypes are associated with aging, mirroring the observations made regarding gut bacterial enterotypes.^26^^,^^31^

### *Candida* enterotype is linked with youth-associated bacterial feature for long-lived individuals

**Figure 4:**
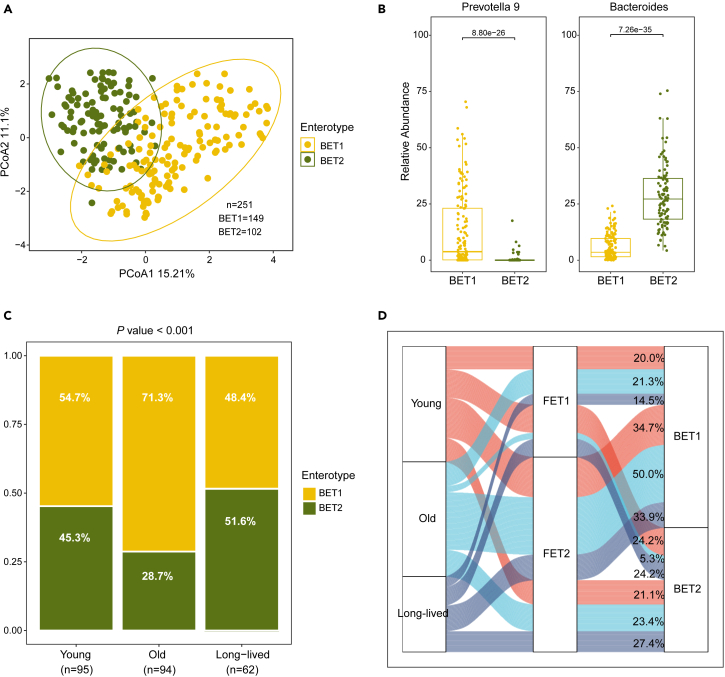
*Candida* enterotype is more likely to coincide with *Bacteroides* enterotype (A) The gut bacterial enterotypes in the gut of aging groups. The bacterial enterotypes were identified using the same strategy as the fungal enterotypes. (B) Relative abundance of the main drivers within each bacterial enterotype. (C) Prevalence of each bacterial enterotype in each age group. (D) The sankey diagram showing the co-occurrences between bacterial and fungal enterotypes within an individual. The *p* values in B were calculated by MaAsLin2 with controlled covariates. The *p* value in C was calculated by chi-square test.

We therefore wondered whether fungal enterotypes were linked with bacterial enterotypes. For these individuals in the whole cohort, we identified two bacterial enterotypes (BET1-2) using the same clustering methods in this study. These microbial enterotypes were characterized by the genera *Prevotella 9* (BET1) and *Bacteroides* (BET2), respectively (Figures 4A and 4B). Approximately 59.3% (*n* = 149) of the individuals exhibited a gut microbiome classified as BET1 (driven by *Prevotella 9*), while the remaining 40.7% (*n* = 102) were categorized as BET2 (*Bacteroides*). In line with the findings in our previous study, we found the BET2 enterotype (driven by *Bacteroides*) is over-represented in the long-lived (51.6%), which is a youth-related feature shared with the young group (45.3%), setting them apart from their older counterparts (28.7%) (Figure 4C; Chi-square test, *p* < 0.001). Therefore, the *Candida-*driven fungal enterotype and *Bacteroides*-driven microbial enterotype represent common signatures shared by long-lived individuals and young individuals, distinctive from general old individuals. Interestingly, we observed that the *Candida* enterotype (FET1) was more likely to coincide with the *Bacteroides* enterotype (BET2) (the link between FET1 to BET2 in Figure 4D) for the individuals of the long-lived group (24.2%) and of the young group (24.2%) in comparison with the situation in the old group (5.3%) (Figure 4D). Together, these data suggest that fungal enterotypes in long-lived people are linked with youth-associated bacterial enterotypes.

### *Candida* enterotype is positively correlated with *Bacteroides* enterotype

We therefore next sought to explore the potential cross-kingdom interactions between gut fungi and bacteria during aging. Sparse Correlations for Compositional Data (SparCC) algorithm was utilized to infer the intra- and inter-kingdom associations at the genus level for young, old, and long-lived groups. A total of 1,959, 1,900, and 1,555 associations were identified in the young, old, and long-lived groups, respectively (|SparCC coefficient| > 0.1, *q* < 0.05, Table S3). Among these links, 305, inter-kingdom associations in the young group, 303 in the old group, and 293 in the long-lived group were identified. Interestingly, inter-kingdom positive correlations were more prevalent in long-lived individuals (26.3%), compared to the proportions observed in the young (22.8%) and old groups (20.1%).

**Figure 5:**
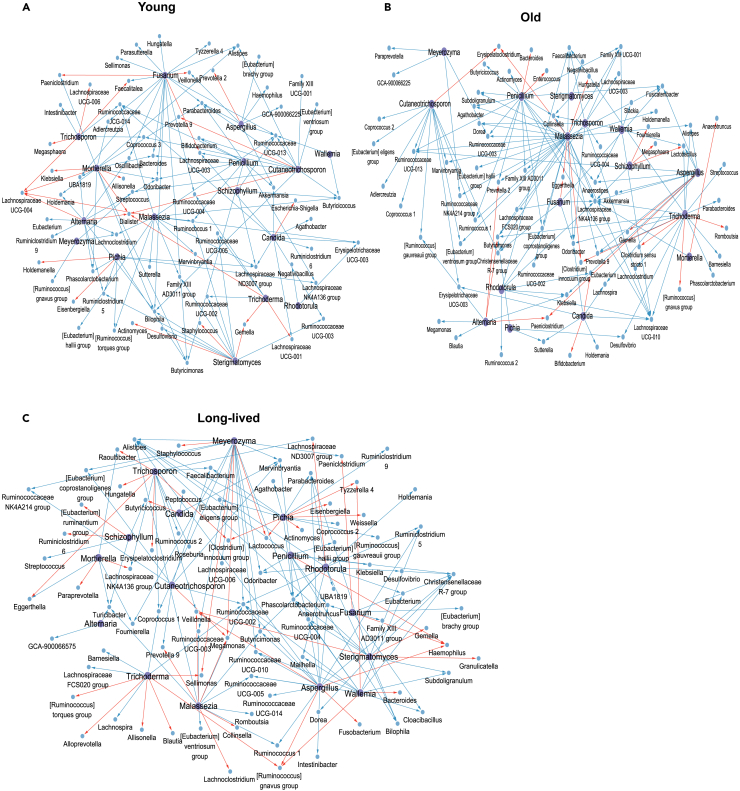
*Candida* enterotype is positively correlated with *Bacteroides* enterotypes for long-lived individuals Co-occurrence networks between fungi and bacteria within the gut were constructed for young (A), old (B), and long-lived (C) groups, respectively. Node color indicates different kingdoms, with purple showing fungi and blue showing bacteria. Red and blue lines indicate positive correlations and negative correlations, respectively. Co-occurrence network analysis was performed by SparCC at the genus level and visualized via Cytoscape.

To figure out the relationships between the fungal enterotypes and bacterial enterotypes within the context of the gut microbiota compositions, we focused on the links between the top 10 drivers for each fungal enterotype and all the bacteria genera. Interestingly, we observed extensive correlations between the top drivers of FET1 (*Candida*) and the top 30 drivers of BET1 (*Prevotella 9*) and BET2 (*Bacteroides)* (Figures 5A–5C). Among these links, the top drivers of FET1, including *Candida*, *Cutaneotrichosporon*, or *Meyerozyma*, were negatively correlated with the dominant taxa of BET1 (*Agathobacte*r, *Coprococcus 3*, *Streptococcus*, *Marvinbryantia*, and *Coprococcus 1*) among three age groups. In contrast, other top drivers of FET1 (*Pichia* and *Cutaneotrichosporon*) displayed positive correlations with *Eisenbergiella* and *Tyzzerella 4* that are abundant in BET2, which were observed only in young and long-live groups. By contrast, the FET2 driver-taxa (*Aspergillus*, *Penicillium*, *Fusarium*, *Malassezia*, or *Rhodotorula*) were negatively correlated with the dominant genera *Parabacteroides*, *Alistipes*, *Ruminococcus 1*, *Butyricimonas*, and *Odoribacter* within BET2 among the aging groups. Notably, these bacteria are well-recognized for their beneficial potential in anti-inflammatory activity, obesity alleviation, and reduction of metabolic dysfunction.^32^^,^^33^ These findings suggest that the taxa enriched in the *Candida* enterotype, which is over-presented in long-lived individuals, are closely associated with the gut bacteria for the *Bacteroides* enterotype. This finding together with the observation of the associations of the prevalence of fungal and bacteria enterotypes across the groups point to a possibility that the *Candida enterotype and Bacteroides* enterotype are two closely related microbiota configurations shared by both long-lived and young individuals.

### Fungal signatures to fingerprint long-lived individuals

**Figure 6:**
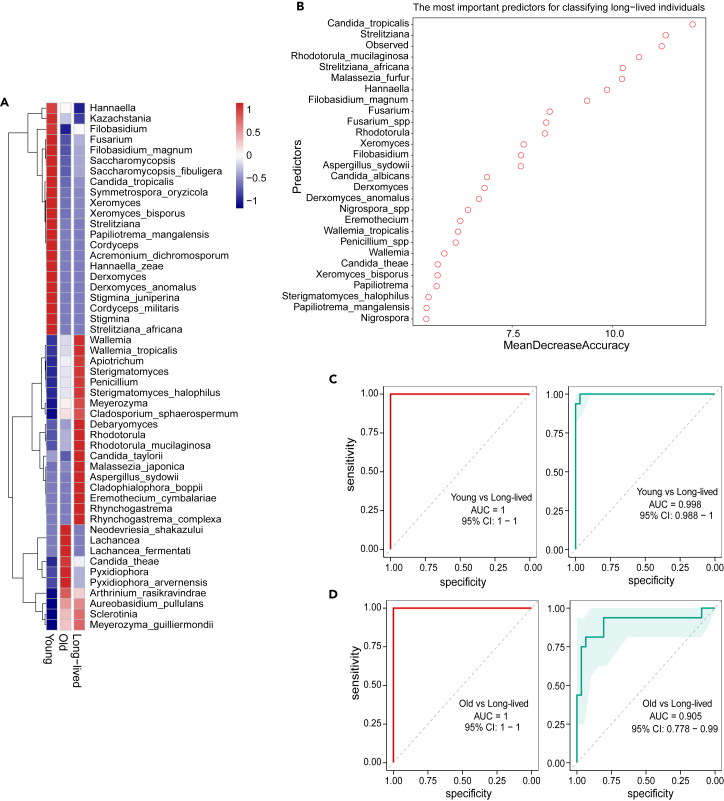
Fungal signatures to fingerprint long-lived individuals (A) Heatmap showing the mean relative abundance of taxa with differential abundances. The differential taxa were determined by MaAslin2. (B) The 28 optimal markers were identified in the random-forest (RF) model by the discovery dataset (*n* = 175). Receiver operating characteristic (ROC) curves for the discovery (red) and validation datasets (green). The AUCs of the validation dataset (*n* = 76) were 0.998 and 0.905 for the comparisons of young versus long-lived cohort (C) and old versus long-lived cohort (D) comparisons, respectively. The 95% confidence intervals (CIs) are shown in red-shaded and green-shaded areas. The error band represents the 95% confidence level for each ROC curve.

The aforementioned discoveries motivated us to investigate what differentiated signatures in the context of gut fungi for long-lived individuals. We conducted differential abundance analysis by MaAsLin2 for the aging groups. When compared to the old group, the young and long-lived individuals showed 79 and 30 differential fungal taxa, respectively (*FDR* < 0.25; Table S3). Among the 30 fungal taxa that were enriched in long-lived populations in comparison with old individuals (Figure 6A), *Debaryomyces*, *Sterigmatomyces halophilus*, and *Rhodotorula mucilaginosa* were reported for their beneficial functions in improving the immune response and maintaining intestinal homeostasis. Additionally, *Penicillium* and *Meyerozyma guilliermondii* were studied for their antifungal and anticancer activities. However, opportunistic fungal pathogens were also identified in this group, such as *Aspergillus sydowii* and *Cladosporium sphaerospermum*. Nonetheless, most of the fungal taxa have not been well studied and their potential roles in the health and disease of the host are almost unknown.^34^^,^^35^^,^^36^^,^^37^^,^^38^^,^^39^^,^^40^ These data highlight that unique fungal features exist in the long-lived group. We further performed machine learning analysis based on Random Forest (RF) to build a mycobiome-based group-classifying model to differentiate long-lived individuals from others. Totally, a combined 28 features (MeanDecreaseAccurary >5) that could effectively discriminate long-lived cohorts from other age groups were identified (Figure 6B). As expected, many of the features that were significantly enriched in long-lived individuals, such as *Wallemia and R*. *mucilaginosa*, were included in the markers of this model. Utilizing this RF-classifier, we could successfully differentiate long-lived individuals from young individuals (areas under the curve [AUC] = 1) and old individuals (AUC = 1) with high accuracy for the discovery cohort (Figures 6C and 6D). For the validation cohort, these markers could also be capable of identifying long-lived individuals from other groups individuals with AUCs of 0.998 and 0.905, respectively (Figures 6C and 6D). These findings indicate that special gut fungal signatures are useful for fingerprinting a long-lived individual.

## Discussion

Comprehensive information on the taxonomic community structure of the gut mycobiome in aging is limited, largely due to small sample sizes or the absence of a sufficiently long trajectory used in mycobiome studies. Using a long trajectory (ages 24–108) cross-sectional analysis, we have uncovered that aging is the primary driver of fungal compositional variation and have identified unique signatures in the gut mycobiome of long-lived individuals. These findings provide insights into the relationship between gut fungal and healthy aging.

Our results highlight the gut mycobiome of long-lived populations exhibited fungal compositional and diversity differences when compared with their young or old counterparts (Figure 2). In line with the findings in the present study, some previous studies also found that aging is closely related to the gut mycobiome.^17^^,^^20^^,^^26^ Wu et al. reported the fungal compositions in the Sardinian cohort of long-lived individuals (*n* = 65, including 22 long-lived people). Interestingly, this work found that the gut fungal community in centenarians was not significantly distinct from those of young or elderly. This inconsistency could be attributed to the variations in geography regions, sample sizes, diets, populations, and age distributions of groups between studies.^18^ Notably, we observed that the fungi in the gut of long-lived individuals were characterized by an increased cumulative relative abundance of core taxa. Given that core fungal taxa have been suggested to be potentially linked to metabolic health,^17^^,^^41^ this feature in long-lived individuals suggests a potential association between core fungal taxa and their longevity. In addition, the long-lived group maintains comparable fungal diversity in the context of the Shannon index and evenness compared with those of other age groups in the fungal community. This co-occurrence suggests that fungal core taxa are associated with maintaining the fungal species diversity during aging, similar to the observations in gut bacterial studies.^42^^,^^43^ In alignment with this notion, a similar trend of decreased number of species of non-core taxa was observed for the gut microbe in our cohort and in other studies,^20^^,^^44^^,^^45^ indicating that non-core taxa are depleted in aging. These findings indicate that the observed gut mycobiota drift toward a unique compositional state seen in long-lived individuals is characterized by an increase of core taxa and a depletion of non-core taxa in the gut mycobiome, which may serve as a useful biomarker for tracking gut mycobiome changes throughout the aging process in humans.

Another striking finding in this study is the association between aging and gut mycobiota enterotypes for long-lived individuals (Figure 3). The enterotype concept, introduced in 2011 to summarize the human gut microbial landscape, is effective in stratifying populations to provide a global overview of the inter-individual variations in gut microbial composition. The association between bacterial enterotypes and host age has been reported in several studies.^20^^,^^30^^,^^31^^,^^46^^,^^47^ However, there is limited knowledge regarding the relationship between fungal enterotypes and aging. In this study, we observed that fungal enterotypes are associated with aging and showed distinctive features in long-lived individuals. The links between *Candida*-driven fungal enterotype and *Bacteroides*-driven microbial enterotype in our cohort are both over-represented in long-lived and young individuals, distinctive from general old people. As the *Bacteroides enterotype* has been identified as a youth-*a*ssociated feature shared between centenarians and young individuals, the findings herein might point to a possibility that fungal-bacterial interactions in the content of enterotype also reflect as a youth-associated feature for long-lived individuals. This hypothesis is supported by the results of co-occurrence analysis that the dominant taxa in *Candida* enterotype *and Bacteroides* enterotype are closely linked within the gut of long-lived participants. It is worth noting that short-chain fatty acids (SCFAs) are the main metabolites produced by the bacteria in the intestine, that have been shown to have multiple effects on *C. albicans* under *in vitro* culture conditions.^48^^,^^49^ In addition, a recent study reported that *Candida* enhances the *in vitro* growth of the strict anaerobes *Bacteroides* spp.^50^

Our findings revealed that *Candida* or *Candida* enterotype is enriched in young and long-lived populations when compared to the elderly group. Nevertheless, the recent study of 3,363 individuals from 16 cohorts across three continents suggests that the elderly group contains a higher proportion of the *Candida* enterotype.^20^ This difference may be attributable to the fact that the cohort used in this study are different in multiple aspects, including geography regions, populations, sample size, and age distribution of individuals of the comparison groups. In particular, this study includes long-lived groups, whereas the work by Lai et al. lacks this group of long-lived individuals. All these variables could affect the enterotypes of the gut mycobiome. Further large-scale exploration considering these factors is warranted to better understand the inter-individual variation in fungal compositions across aging groups. It is noteworthy that *Candida* is most frequently taxa detected in the feces of healthy humans,^28^^,^^51^ and studies have noticed that some *Candida* species also have a beneficial role in human health. For example, *Candida albicans* was found to protect against lethal murine *Clostridium difficile* infections in mice.^52^ However, opportunistic infections of some *Candida* species can occur in individuals with weakened immune systems, such as organ transplant recipients or cancer patients receiving chemotherapy.^4^^,^^16^^,^^53^ As we used the ITS (internal transcribed spacers) sequencing technique to profile fungi in this study, we failed to identify the aging-related *Candida* species at a high resolution. Therefore, further in-depth investigations are needed to identify the taxa at a species level and determine the role of these genera in the gut and during aging.

In this study, the participants had very similar dietary habits (a balanced diet) in our questionnaire. However, we did not accurately assess their diet by a food frequency questionnaire. The influence of diet on the variations of the microbiota compositions for each individual was not evaluated. As previous studies have reported long-term dietary patterns are associated with bacteria enterotypes,^30^^,^^54^ diet might also have influences on the both bacterial and fungal enterotypes for the individuals of the cohort in this study. Furthermore, the aging-associated factors, such as the lifestyle and factors that affect digestion (e.g., tooth loss, and chewing difficulties), also vary during aging. Thus, aging is likely to affect fungal enterotypes in combination with other factors.^20^ Further investigations based on detailed diet information, in particular for long-lived people, are warranted to gain a comprehensive understanding of this issue.

In conclusion, this study highlights the importance of aging in shaping gut mycobiome variations. Additionally, the findings also revealed that the gut mycobiome for long-lived individuals exhibits unique signatures distinctive from other elders, which is characterized by an increase of core taxa and an over-represented *Candida* enterotype. Importantly, these longevity-associated features are also closely linked to gut bacterial compositions and could serve as biomarkers to effectively differentiate long-lived people from others. These findings provide novel insights for a better understanding of the relationship between gut microbiota and aging.

### Limitations of the study

In this study, we report the gut mycobiome signatures for long-lived individuals. Our findings suggest that the gut mycobiome develops with aging, and long-lived individuals possess unique fungal signatures. However, this study does have several limitations. First, the participants in the cohort were recruited from a single geographical region (Guangxi, China). This may limit the generalizability of our findings to other populations or areas with different lifestyles, diets, and genetic backgrounds. Second, as our study is cross-sectional, it does not provide insights into changes in the gut mycobiome over time. Longitudinal studies are needed to confirm our findings and to better understand the dynamics of the gut mycobiome during aging. Third, while we controlled for several potential confounders, such as sex, BMI, lifestyles, and disease, other unmeasured variables (e.g., diet, medication use, and environmental factors) may have influence on the gut mycobiome compositions. In the end, we cannot establish causality between the identified fungal signatures and aging. Experimental and interventional studies are required to determine whether and how these fungal signatures contribute to health and aging. In conclusion, while our study provides valuable insights into the gut mycobiome of long-lived individuals and highlights unique fungal signatures associated with aging, it also underscores the need for further research to address these limitations and expand upon our findings.

## STAR★Methods

### Key resources table

**Table:** 

REAGENT or RESOURCE	SOURCE	IDENTIFIER
**Biological samples**		

Stool samples of participants	Guangxi Province, China	N/A

**Deposited data**		

UNITE (v8.3)	Abarenkov et al.^55^	https://unite.ut.ee/repository.php.
Sliva_132	Quast et al.^56^	https://www.arb-silva.de/
Raw sequence data	This paper	NCBI: PRJNA1062552

**Software and algorithms**		

QIIME2 (v2020.2)	Bolyen et al.^57^	https://qiime2.org/
aPCoA (v1.3)	Shi et al.^58^	https://github.com/YushuShi/aPCoA
Vegan (v2.6–4)	N/A	https://github.com/vegandevs/vegan
Phyloseq (v1.44.0)	McMurdie et al.^59^	https://github.com/joey711/phyloseq
microbiome(v1.22.0)	N/A	https://github.com/microbiome/microbiome
MaAsLin2 (v1.14.1)	Mallick et al.^60^	https://github.com/biobakery/Maaslin2
ade4 (v1.7-22)	N/A	https://github.com/adeverse/ade4
FastSpar (v1.0.0)	Watts et al.^61^	https://github.com/scwatts/fastspar
randomForest (v4.6-14)	N/A	https://github.com/cran/randomForest
R(v4.1.2)	N/A	https://www.rproject.org/
Custom code and script used in this study	This paper	https://github.com/LixiaPu/Long-lived-gut_mycobiome

### Resource availability

#### Lead contact

Further information and requests can be directed to Prof. Shuai Wang (wangshuai@caas.cn).

#### Materials availability statements

The study did not generate new unique reagents.

#### Data and code availability

The raw sequence data are available in the Sequence Read Archive (SRA) of the U.S. National Center for Biotechnology Information (NCBI) under BioProject PRJNA1062552. All code used in this study is available in GitHub (https://github.com/LixiaPu/Long-lived-gut_mycobiome) or can be found on websites of corresponding software packages. Any additional information required to reanalyze the data reported in this paper is available from the lead contact upon request.

### Experimental model and study participant details

#### Ethics approval and consent to participate

This study was approved by the ethics committee of AIage Life Science Corporation (ref. no. 2019–001) and the ethics committee of the First Affiliated Hospital of Guangxi Medical University (ref. no. 2020-KT-050). All participants signed an informed consent agreement before donating their fecal samples.

#### Study participants and stool sample collection

The current study was based on the Gut Microbiome and Longevity Project (GMLP). The dataset here involved 251 participants including 62 long-lived individuals (aged 95 to 108 years; average 101 ± 3.03), 94 old adults (aged 62 to 85 years; average 69.6 ± 5.53), and 95 young adults (aged 24 to 44 years; average 34.5 ± 4.66). All participants were community-dwelling and permanently lived in Guangxi province, China. Detailed information assessed in the present study was collected through a face-to-face questionnaire, including demographics, medical history, dietary habits, drug use, and lifestyle (Table 1). Participants were excluded from enrollment if they had exhibited severely impaired cognition (as determined by a suitable Mini-Mental test assessing cognitive ability and the ability to respond to the questionnaire), serious chronic diseases (such as diabetes), acute/chronic intestinal diseases use of antibiotics (antibacterial or antifungal), or taking any probiotics (drinking yogurt or other probiotic supplements) in the past 3 months, or were diagnosed malignant neoplasia or acute infectious diseases.

Stool samples were collected by the participants at home. Participants were provided with DNA collection kits with a proprietary chemical DNA stabilizer to maintain DNA integrity at ambient temperature after collection.^62^ After passing stool, a spoon was used to collect stool samples by scraping off the outer layer of solid feces and collecting the central part into the tube. Samples were divided into aliquots of 200 mg and stored at −80°C until used in the laboratory.

### Method details

#### DNA extraction and PCR amplification

Microbial DNA was extracted from all fecal samples using a Fecal Nucleic Acid Extraction Kit (Surbiopure) according to the manufacturer’s protocols. The ITS1 hypervariable regions of the fungal ITS rRNA gene were amplified with primers ITS5-1737F (5′-GGAAGTAAAAGTCGTAACAAGG-3′) and ITS2-2043R (5′-GCTGCGTTCTTCATCGATGC-3′) and barcodes. PCR amplification system contained a 15 μL mixture of Phusion High-Fidelity PCR Master Mix (New England Biolabs), 0.2 μM of each primer, and 10 ng target DNA. The PCR reactions were conducted using the following program: 1 min of initial denaturation at 98°C, followed by 30 cycles of 10 s at 98°C, 30s for annealing at 50°C and 45s for elongation at 72°C and a final extension at 72°C for 5 min. The resultant PCR products were extracted from a 2% agarose gel and further purified using a Qiagen Gel Extraction Kit (Qiagen, Germany) according to the manufacturer’s protocol. Then the ITS libraries were generated with NEBNext Ultra IIDNA Library Prep Kit (Cat No. E7645) according to the manufacturer’s instructions and quantified using a Qubit@ 2.0 Fluorometer (Thermo Scientific). Amplicon size was determined using a Bioanalyzer (Agilent).

#### Sequencing and data processing

The constructed libraries were sequenced on an Illumina NovaSeq 6000 platform according to the standard protocol and 250 bp paired-end reads were produced. QIIME2 was used for the downstream analysis (QIIM2-2020.2).^57^ The demultiplexed ITS sequences were filtered, denoised, and grouped into amplicon sequence variants using DADA2 implemented in QIIME2 with default parameters. Overall, a total of 18.5 million reads were generated from all samples to represent the final high-quality nonchimeric reads (with a median of 73,621 reads per sample; Table S5). A naive Bayesian classifier was trained using the QIIME2 q2-feature-classifier plugin on the UNITE reference database (v8.3) with ASV-defined 99 identity.^55^ Taxonomy was assigned using the naive Bayesian classifier.

To compare the gut microbiome composition of the three age groups, we also analyzed the 16S rRNA sequencing data for these individuals.^26^ Briefly, sequences were merge-paired, quality-filtered, and analyzed using QIIME2 (v2020.2). As described above, we used the DADA2 denoised-paired plugin in QIIME2 to process the fastq files. The taxonomies of ASVs were subsequently determined using the Naive Bayes classifier trained on the Sliva_132 99% reference database.^56^

#### Fungal diversity and covariates effect size analysis

PCoA was conducted to display microbial distance based on unweighted UniFrac distances using the phyloseq package. Furthermore, covariate-adjusted principal coordinates analysis (aPCoA) was used to adjust for confounders (Age, Sex, BMI, Smoking status, Alcohol drinking, Tea drinking, and Hypertension) and for visualization when comparing mycobiome differences between age groups.^58^ The effect size and significance of each covariate were determined using the ‘envfit’ function in ‘vegan’ by comparing the difference in the centroids of each group relative to the total variation. Ordination was performed using the NMDS method based on Bray-Curtis dissimilarity. The significance value was determined based on 999 permutations. All *p* values derived from envfit were adjusted for multiple comparisons using *FDR* adjustment in R.

#### Richness, core taxa, and differential taxa analysis

Alpha diversity was estimated by Observed number, Shannon index, and Pielou’s evenness index using the ‘estimate_richness’ and ‘evenness’ functions in the ‘phyloseq’ package.^59^ Core taxa were calculated by the R package “microbiome” using the cutoff: present in at least 85% of the individuals with a minimum relative abundance higher than 0.01%. Significant differential taxa were determined by MaAsLin2 with adjusted covariates (genus and species level) and the default parameters (maximum percentage of samples NA in the metadata 10%, minimum percentage relative abundance 0.01%, *p* < 0.05, *q* < 0.25).^60^

#### Enterotype analysis

The gut fungi and bacteria components of the samples were clustered by following a previously published protocol for partitioning around medoid (PAM)-based clustering using Jensen–Shannon distance at the genus level.^30^ To decrease noise, only genera present in more than 20% of the samples were included. The optimal number of clusters in PAM was estimated using the Calinski-Harabasz index. Between-class analysis (BCA) was performed to validate the clustering results and identify the drivers for each cluster, and the clustering results were then visualized through principal coordinates analysis (PCoA) by the “dudi.pco” function in the ‘ade4’ package in R.^63^ Moreover, MaAsLin2 analysis was also used to assess the differential relative abundance between enterotype drivers with covariates adjusted.

#### Correlation network analysis

Only genera that were present in more than 20% of samples were included in the correlation analysis. The SparCC algorithm, wrapped in the FastSpar application, was used to estimate the inter- and cross-kingdom taxonomic associations based on the raw reads count tables.^61^^,^^64^ The analysis with default parameters and 1000 bootstraps was used to infer *p* values.

#### Machine learning algorithm

A random forest (RF) classification model was used to identify the fungal features that are able to differentiate the three age groups.^65^ The model was built with the rarefied ASV count data and species richness, and ASVs present in <1% of all samples were filtered out for further analysis. All samples in our study were randomly partitioned into a training dataset and a validation dataset with a proportion of 7:3 for the samples. For the RF classification model, the training dataset was used to pick out the optimal set of fungal markers to predict classification by using 10-fold cross-validation (replicates = 5) (The R package randomForest 4.6–14) with default parameters. The point with the minimum cross-validation error was used as the cutoff point, and the first 28 features were selected as the mycobiome markers based on the ranked value of MeanDecreaseAccuracy. The identified optimal set of the top 28 features was applied to test the capability of prediction for the training data and validation data in the final “bagged” random forest classifier as evaluated by the receiver operating characteristic (ROC) curve (pROC package v1.18.0). The area under the ROC curve (AUC) was used to designate the ROC effect.

### Quantification and statistical analysis

Bioinformatic analysis was described in the method details section. All statistical analyses were performed using the R software (v4.1.2). The comparisons of taxa abundance, alpha diversity, and distance between groups or enterotypes were performed using the MaAsLin2. Discrimination among groups was detected by the Adonis method in vegan in R. All permutation tests were conducted using 999 permutations. A one-way ANOVA with Tukey’s post hoc test was used to evaluate the differences between the PCo1 and PCo2 of the groups.

## Supplemental information

Document S1. Figures S1–S6

Table S1. Summary of the metadata for the aging groups (n = 251), related to Table 1

Table S2. The effect size of metadata variables on the gut mycobiome variations, related to Figure 1C

Table S3. Correlations between the gut fungal and bacterial components in three age groups, related to Figure 5

Table S4. The taxa with differential abundances in MaAslin2 analysis, related to Figure 6A

Table S5. The statistics of the sequencing datasets used in this study, related to STAR Methods
