# Supplementary material for: The gut mycobiome signatures in long-lived populations
Source: iScience. 2024 Jun 28;27(8):110412. doi: 10.1016/j.isci.2024.110412 (PMC11284699; doi:10.1016/j.isci.2024.110412)
Supplement: Document S1. Figures S1–S6 [file mmc1.pdf]

iScience, Volume *27*

Supplemental information

The gut mycobiome signatures

in long-lived populations

Lixia Pu, Shifu Pang, Wenjie Mu, Xiaodong Chen, Yang Zou, Yugui Wang, Yingying Ding, Qi Yan, Yu Huang, Xiaochun Chen, Tao Peng, Weifei Luo, and Shuai Wang

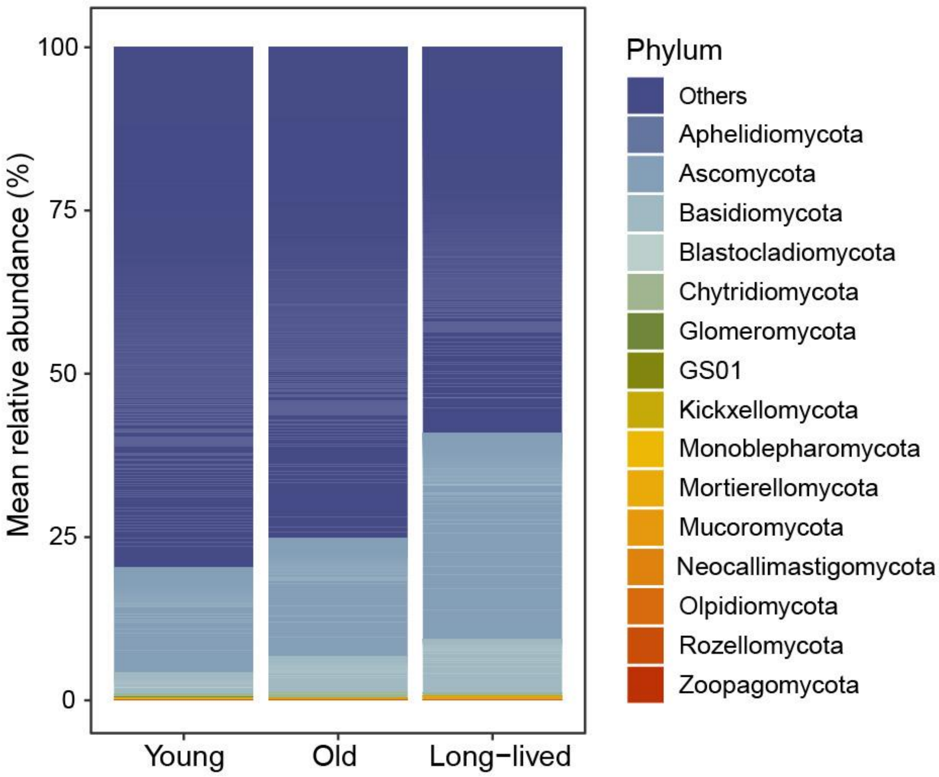

Figure S1. The mean relative abundance of each phylum within each aging group, related to Figure 1A.

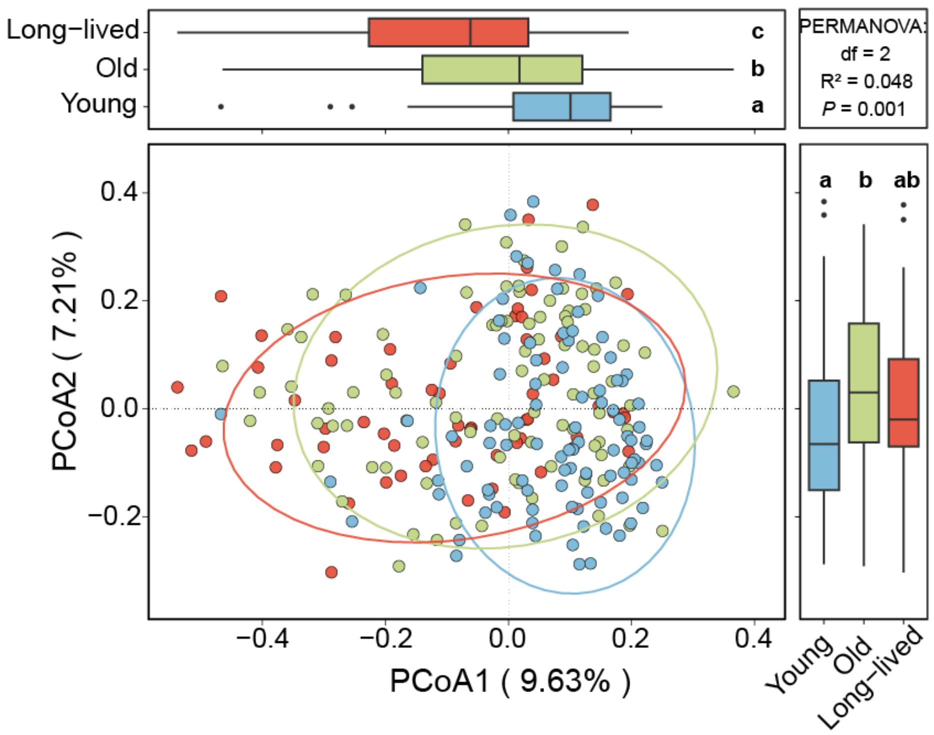

Figure S2. Variation of the gut bacterial components among aging groups based on Bray-Curtis distances (Adonis test, P = 0.048, R^2 = 0.001), related to Figure 1B. Boxes with no common letters indicate significant differences in one-way ANOVA with two-sided Tukey's post hoc test (P < 0.05).

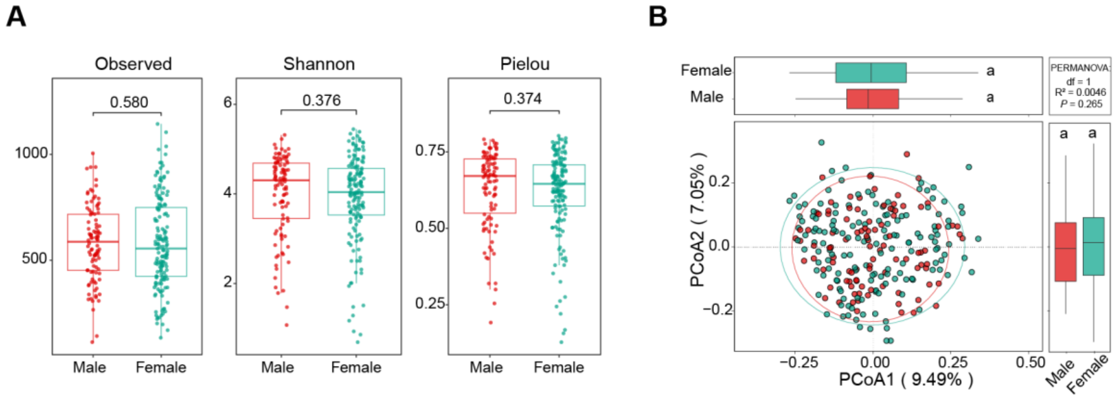

Figure S3. There was no significant difference in terms of mycobiome community structure between males and females, related to Figure 1C. (A) Comparison of the gut mycobiome α-diversity, as indicated by Observed, Shannon, and Pielou's evenness indices between males (n = 98) and females (n = 153). The *P* values were calculated by MaAsLin2 with controlled covariates (Age, BMI, Tea drinking, Alcohol drinking, and Hypertension). (B) Variation in the gut mycobiome components between males (n = 98) and females (n = 153) based on Unifrac distances at ASV level (Adonis test, R^2 = 0.0046, *P* = 0.256). Data in boxes with no common letters are significantly different in one way ANOVA with two-sided Tukey's post hoc test (*P* < 0.05).

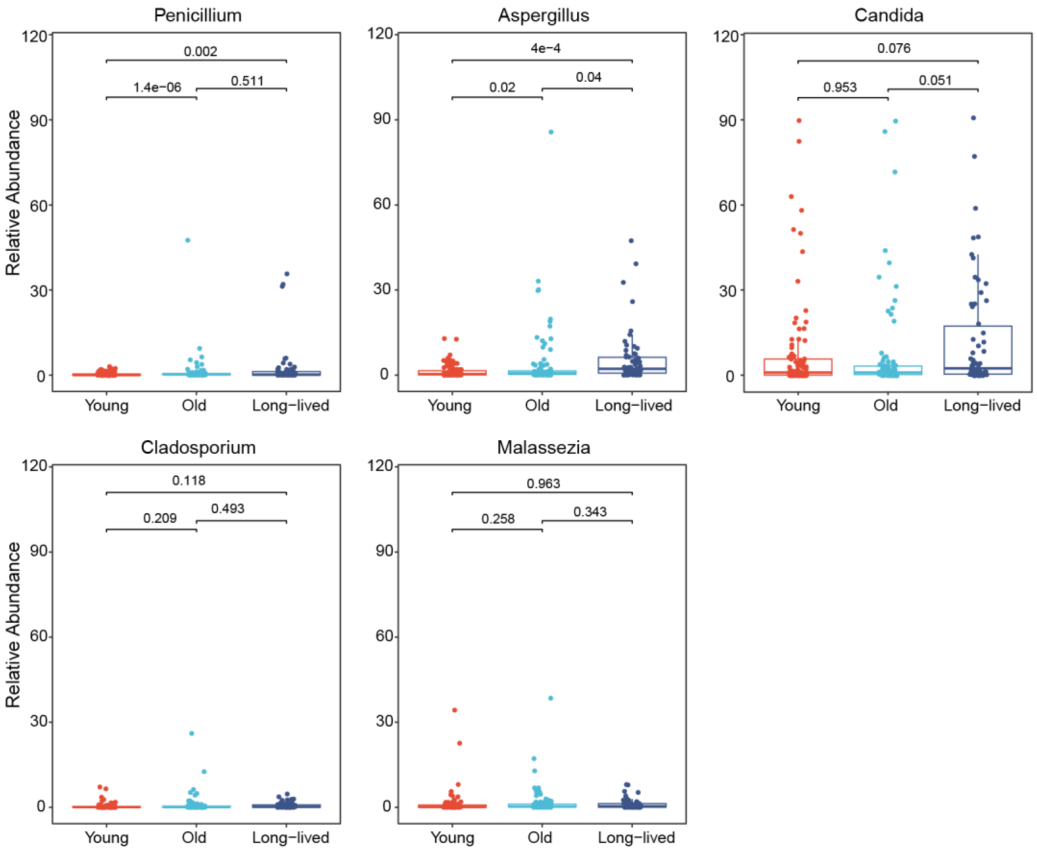

Figure S4. Comparisons of core taxa among different aging groups, related to Figure 2A. The *P* values were calculated by MaAsLin2 with controlled covariates (Sex, BMI, Tea drinking, Alcohol drinking, and Hypertension).

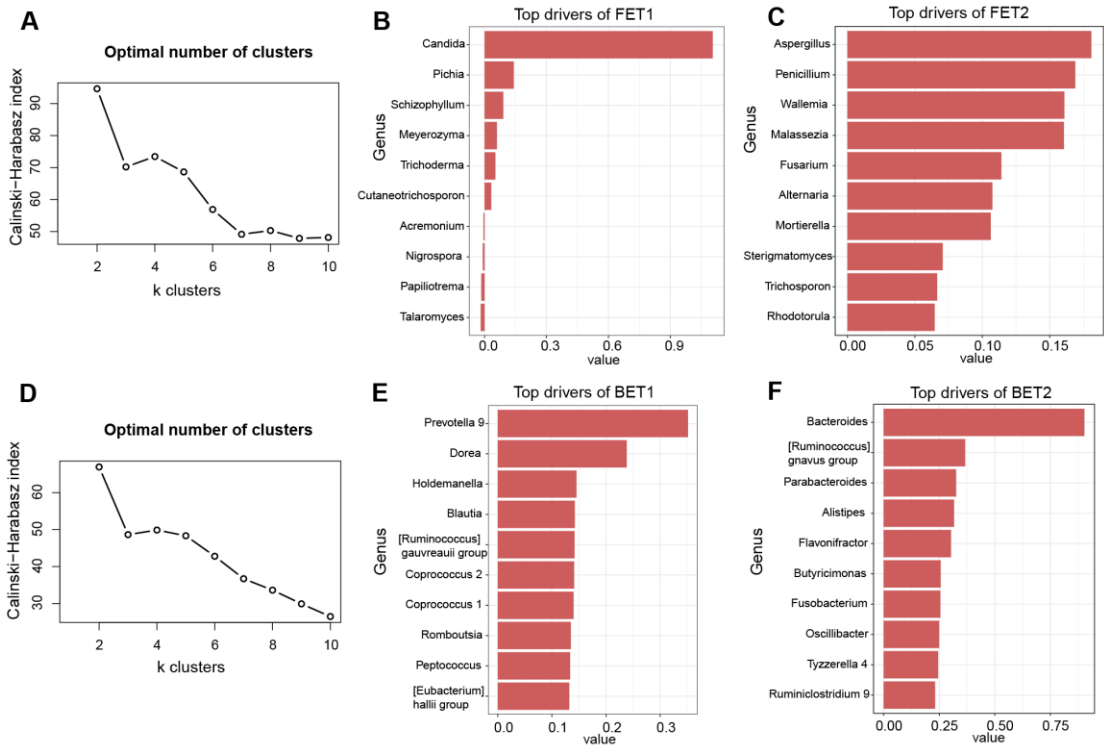

Figure S5. The identified fungal and bacterial enterotypes for all the samples, related to Figure 3 and Figure 4. Calinski-Harabasz index for the Jensen–Shannon distance (JSD) and partitioning around medoid (PAM) indicates that the optimal number of the gut mycobiome. (A) and the gut microbiome clusters in the cohort. The top 10 drivers of *Candida*-enterotype (B), *Aspergillus*-enterotype (C), *Prevotella 9*-enterotype (E), and *Bacteroides*-enterotype (F) in the cohort are listed, respectively. The values of the x-coordinate indicate the contribution of each variable to the enterotype in between-class analysis (BCA).

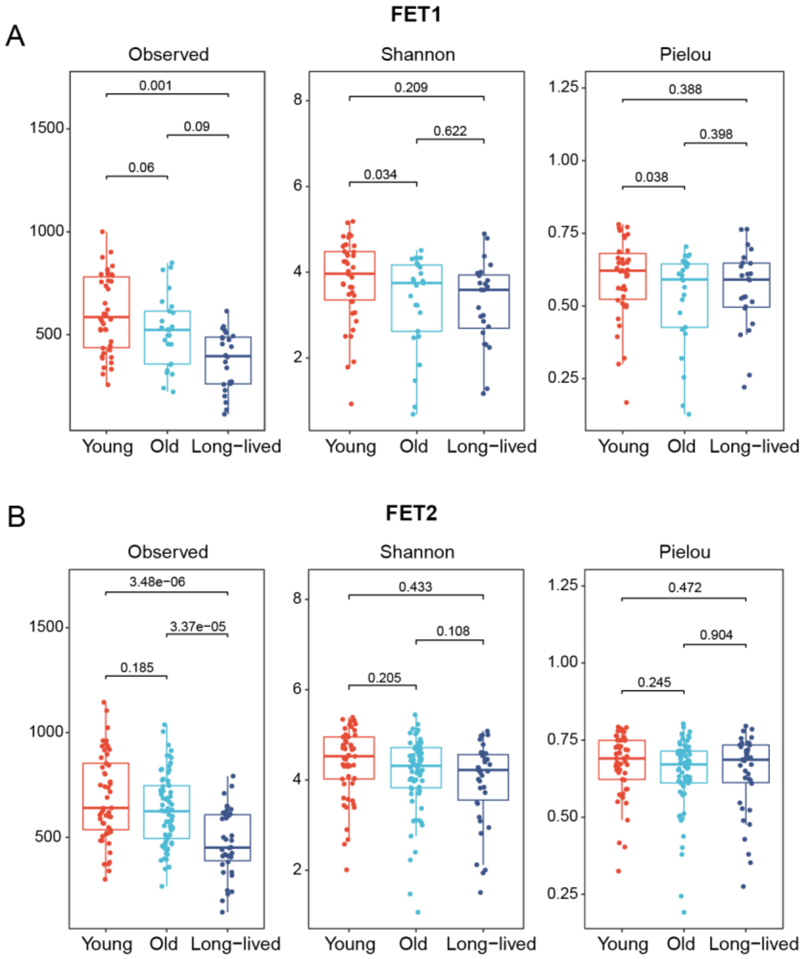

Figure S6. Comparisons of α-diversity among the aging groups, related to Figure 3. Observed, Shannon and Pielou's evenness indices are shown in the enterotypes of FET1 (A) and FET2 (B).
